# Contemporary Trainee Knowledge of Autism: How Prepared Are Our Future Providers?

**DOI:** 10.3389/fped.2019.00165

**Published:** 2019-04-26

**Authors:** Kristine Austriaco, Inmaculada Aban, James Willig, Michele Kong

**Affiliations:** ^1^School of Medicine, University of Alabama at Birmingham, Birmingham, AL, United States; ^2^University of Alabama at Birmingham School of Public Health, Birmingham, AL, United States; ^3^Department of Medicine, University of Alabama at Birmingham, Birmingham, AL, United States; ^4^Department of Pediatrics, University of Alabama at Birmingham, Birmingham, AL, United States

**Keywords:** autism, sensory dysregulation, sensory processing disorder, medical education, ASD, communication, pediatric

## Abstract

**Background:** Over the last several decades, the prevalence of Autism Spectrum Disorder (ASD) has continued to increase, creating a unique challenge for general physicians who are likely to encounter these patients in their practice. The primary aim of this cross-sectional study design was to identify potential knowledge gaps that were present among medical students and pediatric trainees (interns, residents, and fellows) particularly during the management of a sick child with ASD.

**Methods:** A 23-question online survey was developed and distributed to medical students and pediatric trainees at a tertiary children's hospital affiliated with a medical school.

**Results:** Medical students and pediatric trainees reported a low general knowledge of ASD and were unfamiliar with sensory issues that are often present in these children. Increased discomfort and insufficient didactic and clinical training for providing care to children with ASD during an acute illness were also identified. Both medical students and trainees reported the need for increased education and training, preferentially via patient interaction and small group-based learning. We found that as education/training levels increased, participants perceived increased comfort, and knowledge in managing an ill child with ASD.

**Conclusions:** A perceived knowledge gap and discomfort is present amongst medical students and pediatric trainees on the management of children with ASD. Across all education levels, awareness for sensory dysregulation in ASD children is low. Education programs using direct patient interaction and small group learning were the preferred training modalities to learn how to provide optimal care for children with ASD.

## Background

The prevalence for Autism Spectrum Disorder (ASD) has continued to increase over the past two decades. According to a recent report by the Centers for Disease Control and Prevention (CDC), 1 in 59 children are diagnosed with ASD, with boys being 4 times more likely to be diagnosed than girls (1). The age of diagnosis is typically around 4 years of age, but a child as young as 12 months old can be diagnosed (2). ASD is often characterized by repetitive mannerisms, and deficits in both communication and social behaviors (3). Much of the deficits in communication stem from the delay in the onset of speech, poor understanding of the words being spoken to them, and the inability to use speech appropriately in social situations, such as not answering the question being asked or using an improper tone of voice (4). In addition, children with ASD often have difficulty interpreting sounds or visual information, which may lead to a decrease or lack of response to normal social cues, further hindering the formation of relationships with others (5). These children typically have a difficult time adjusting to new surroundings and stimuli, often favoring their everyday, predictable routines. Even with these common characteristics, managing children with ASD, particularly when they are ill can be difficult as no two children present the same way (6). It is critical that primary care physicians recognize core characteristics of children with ASD as it may translate to earlier diagnosis and intervention, as well as appropriate management when they are unwell (7, 8).

During an acute illness, healthcare providers often face a unique challenge in managing patients with ASD because of their core characteristics. The limited verbal communication, impaired social interaction, repetitive behaviors, aggression, and sensory sensitivities can become barriers to medical diagnosis and management during the visit. In addition, interruption in the child's routine, the new environment and exposure to a multitude of new faces, and encounters may further exacerbate the irritability that even typical children have during an acute illness (9). Environmental stimuli such as the bright lights, noise, smell, and the fast pace of the acute care clinic or Emergency Room (ER) can further compound the sensory processing disorders that many children with ASD have. It is critical that a physician distinguishes the baseline or exacerbated behavior of an acutely ill child with ASD from new medical symptoms that led to the visit in order to prevent misdiagnosis and provide the appropriate management (10). This is particularly challenging in an ER or acute care setting whereby the physician is unlikely to have a previous relationship with the child or family and therefore limited baseline knowledge of the patient or limited insight into how acute illness may affect behavior in children with ASD.

Our overarching hypothesis was that medical students and pediatric trainees would report a gap in their general knowledge of ASD, especially when encountering the sick child with ASD.

## Methods

### Setting, Participants and Study Design

All medical students at The University of Alabama at Birmingham (UAB) Medical School and pediatric residents at Children's of Alabama were eligible to participate and invited to complete a survey (via www.surveymonkey.com). One hundred and ninety one medical students and pediatric residents responded to the call for participation and were enrolled in this cross-sectional study. Non-participation was likely due unawareness to the call for participation and time constraint. The survey was distributed via emails, with 2 follow-up emails over a 3-month period. This population was chosen to allow assessment of perceived knowledge base in ASD across training levels (medical students; pediatric interns, residents and fellows).

### Participant Variables and Survey Questions

Participant variables collected included level of training for medical students (year 1–4) and pediatric trainees (intern, resident, fellow), trainee age range (21–30, 31–40), ethnicity (Caucasian, African American, Hispanic, Other) and gender (male, female). A 23-question survey (Appendix) with questions ranging from general knowledge of children with ASD, including methods of communication, sensory sensitivities, perceived importance for understanding ASD behavior, perceived comfort level in providing care, with focus on differences during acute illness, and perceived adequacy of training in ASD. Several questions required participants to rate their answers on a scale of 1 to 10, with 1 being the lowest score and 10 being the highest score.

### Statistical Methods and Analysis

Descriptive statistics were utilized to summarize data including counts and percentages for categorical variables and means and standard deviations for continuous variables. Trends and association in responses and education status and gender were analyzed using Fisher's exact test for categorical responses and Wilcoxon Test for responses requiring rating between 1 and 10. Responses of primary interest were: perceived best method of communication with children with ASD, perceived importance of daily behavior and daily routine, comfortability of treating a child with ASD who presents with an acute illness, the best method to communicate with children with ASD who present with an acute illness, and the perceived best method of education regarding this topic. For the purpose of comparing educational and training level, participants were either in the medical student group or pediatric trainee group. This was done to ensure an adequate amount of data to enable comparisons. All analyses were done utilizing the SAS 9.4 software program. Any *p* value found to be < 0.05 was deemed significant.

## Results

One hundred and ninety one survey responses were collected and recorded. Demographics of the participants can be found in Table 1. Most of the survey respondents were first year medical students, ages 21–30 years old, and Caucasian. The ratio of men to women surveyed varied on education and training level.

**Table 1:** Demographics.

**Parameters**	**MS1**	**MS2**	**MS3**	**MS4**	**Pediatric intern**	**Pediatric resident**	**Pediatric fellow**	**Overall (*N*)**
Count (%*N*)	64 (33.51)	29 (15.18)	36 (18.85)	24 (12.57)	9 (4.71)	13 (6.81)	16 (8.38)	191
**Age**								
21–30	64 (35.96)	28 (15.73)	35 (19.66)	24 (13.48)	8 (4.49)	12 (6.74)	7 (3.93)	178
31–40	–	1 (7.69)	1 (7.69)	–	1 (7.69)	1 (7.69)	9 (69.23)	13
**Ethnicity**								
Caucasian	55 (34.16)	23 (14.29)	31 (19.25)	18 (11.18)	7 (4.35)	11 (6.83)	16 (9.94)	161
African-American	1 (16.67)	3 (50.00)	1 (16.67)	–	–	–	1 (16.67)	6
Asian	8 (30.77)	5 (19.23)	6 (23.08)	5 (19.23)	1 (3.85)	1 (3.85)	–	26
Hispanic	1 (20.00)	1 (20.00)	–	1 (20.00)	1 (20.00)	1 (20.00)	–	5
Other^*^	–	–	–	–	1 (100.00)	–	–	1
**Gender**								
Male	24 (30.38)	11 (13.92)	13 (16.46)	14 (17.72)	3 (3.80)	6 (7.59)	8 (10.13)	79
Female	40 (36.36)	18 (16.36)	22 (20.00)	10 (9.09)	6 (5.45)	6 (5.45)	8 (7.27)	110

* Other means ethnicities not already included.

*MS, medical student*.

### Cross-Sectional Survey Data

#### General Knowledge of ASD

The perceived general knowledge of ASD was low for both medical students and pediatric trainees. More than 85% of all responders rated their general knowledge of ASD to be less than somewhat informed (Table 2). However, with increased training level, a higher proportion of pediatric trainees stated that they have more general knowledge compared to medical students (*p* = 0.0494).

**Table 2:** Perceived general knowledge of ASD.

	**No information**	**Limited information**	**Somewhat informed**	**Very informed**
Medical students	2.61%	42.48%	47.06%	7.84%
Pediatric trainees	0%	21.05%	65.79%	13.16%

#### Interaction With Children With ASD

Medical students had significantly less interaction with children with ASD when compared to pediatric trainees (39.5% limited to no interaction vs. 83.0% monthly, weekly or daily interaction, *p* < 0.0001). In this cohort of participants, all pediatric trainees had their interaction in a medical setting, while medical students varied on interaction setting (41.4% medical, 34.5% familial, 24.1% work other than medical).

#### Managing a Well-Child With ASD

Medical students rated the importance of understanding baseline ASD behavior lower when compared to pediatric trainees (8.41 ± 1.24 vs. 9.05 ± 1.06 on a 1–10 scale, *p* = 0.004). There was no significant difference found in the perceived importance of understanding the daily routine of ASD children for medical students and pediatric trainees (8.73 ± 1.32 vs. 8.92 ± 1.28 on a 1–10 scale, *p* = 0.338). As a whole, females participants rated understanding ASD behavior higher than their male counterparts (8.79 vs. 8.16 on a scale of 1–10, *p* = 0.005). They also had a higher rating for understanding the importance of ASD routine (8.95 vs. 8.48 on a scale of 1–10, *p* = 0.0107), as well as discussing the child's routine with the family (8.87 vs. 8.33 on a scale of 1–10, *p* = 0.0028).

**Figure 1:**
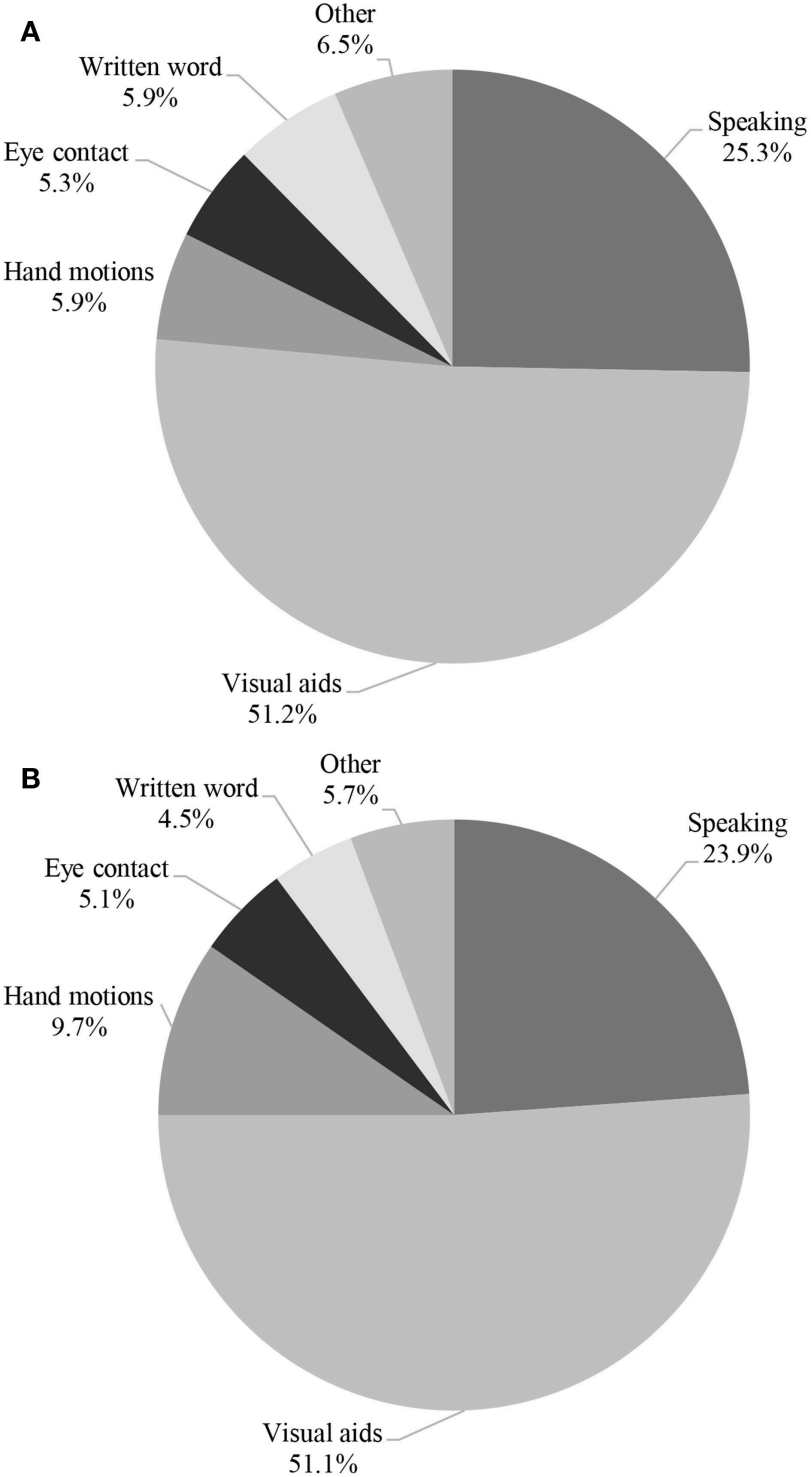
Perceived Best communication method with children with ASD at baseline **(A)** and with **(B)** an Acute Illness.

In terms of communication, medical students and pediatric trainees selected visual aids and hand motions, followed by verbal communication, eye contact and written words for what they *perceived* to be the primary method of communication that children with ASD use [126 (30.1%) visual aids, 124 (29.6%) hand motions, 76 (18.1%) verbal communication, 32 (7.6%) eye contact, 46 (11.0%) written word, and 15 (3.6%) ‘other’]. Other responses included using sounds and gestures, an iPad program, and art. However, for best method of communication to use *with these* children, participants chose visual aids (87, 51.2%), verbal communication (43, 25.3%), hand motions (10, 5.9%), written word (10, 5.9%), eye contact (9, 5.3%), and other (11, 6.5%), respectively (Figure 1A). Other responses included ‘following the child's lead’ and singing.

#### Managing the Sick Child With ASD

More pediatric trainees have provided care to children with ASD compared to medical students (73.7% vs. 15.7%, *p* < 0.0001), with in-patient hospital setting being the most common location for the encounter. Overall, both medical students and pediatric trainees were uncomfortable with providing care to a sick child with ASD (6.63 ± 1.55 vs. 4.61 ± 2.19 on a scale of 1–10, *p* < 0.0001). The medical students and pediatric trainees perceived hand motions, visual aids, verbal communication, eye contact, and written words to be the primary method of communication utilized by sick ASD children [124 (34.4%) hand motions, 108 (29.9%) visual aids, 62 (17.2%) verbal communication, 28 (7.8%) eye contact, 25 (6.9%) written word, and 14 (3.9%) other, respectively]. Other responses included talking to the parents and placing the child in the most familiar environment as possible. A similar trend was noted in the best method to communicate *with* them during an acute illness when compared to communication with a well-child [90 (51.1%) visual aids, 42 (23.9%) verbal communication, 17 (9.7%) hand motions, 9 (5.1%) eye contact, 8 (4.5%) written word, and 10 (5.7%) other; Figure 1B].

#### Sensory Dysregulation

**Figure 2:**
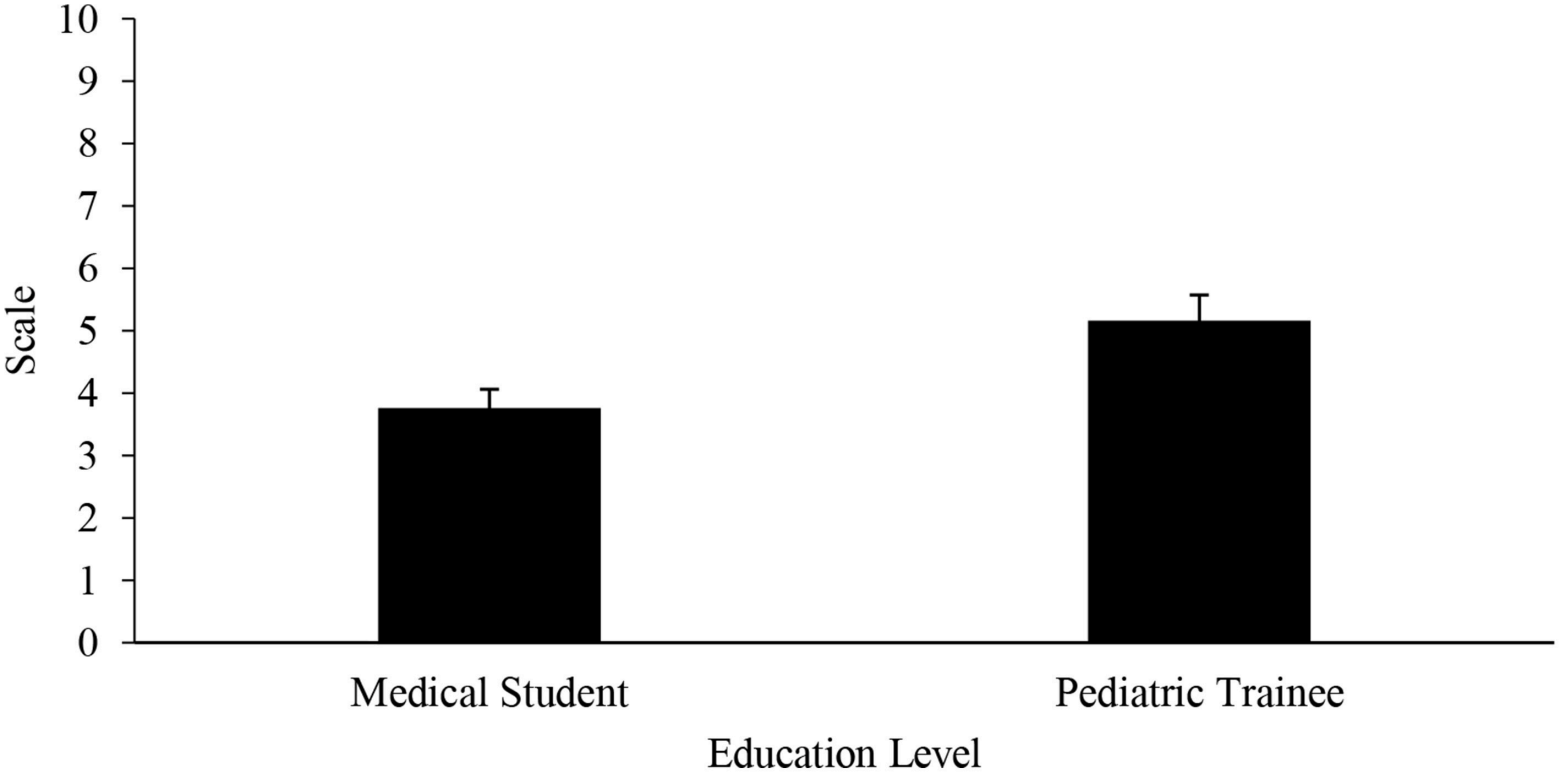
Understanding of sensory dysregulation in children with ASD.

**Figure 3:**
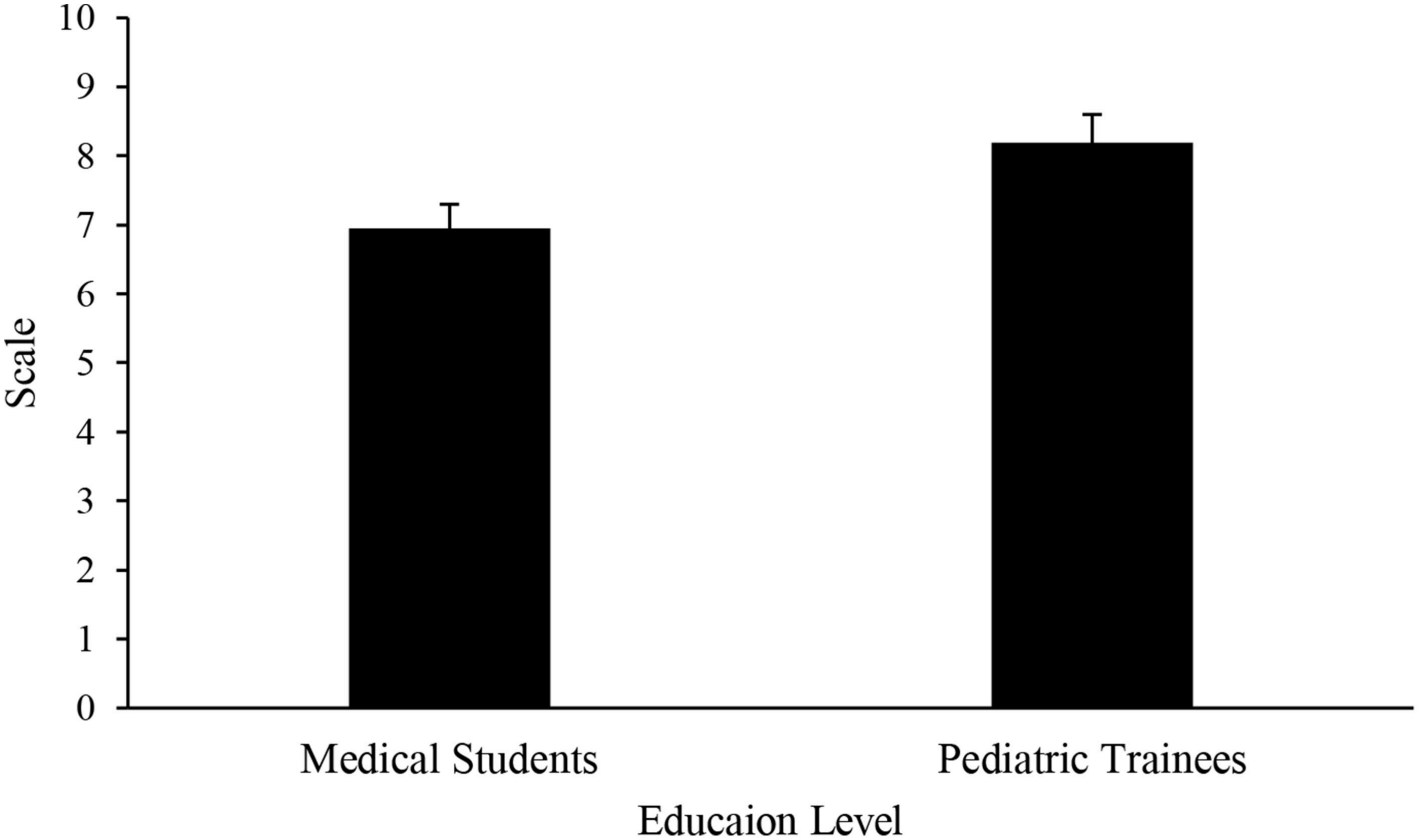
Perceived frequency of sensory dysregulation in children with ASD.

Medical students had a significantly lower rating in the understanding of the sensory issues that can occur in children with ASD when compared to pediatric trainees (3.76 ± 2.54 vs. 5.16 ± 1.83 on a scale 1–10, *p* = 0.0007; Figure 2), but both education groups scored their understanding low overall. Medical students also believed that children with ASD experience sensory dysregulation less frequently than pediatric trainees (6.95 ± 1.55 vs. 8.19 ± 1.22 on a 1–10 scale, *p* < 0.0001; Figure 3). Both medical students and pediatric trainees felt that it was important to elicit information regarding a child's routine when he or she is presenting with an acute illness (8.68 ± 1.44 vs. 8.61 ± 1.55 on a 1–10 scale, *p* = 0.9945).

#### Learning About ASD

**Figure 4:**
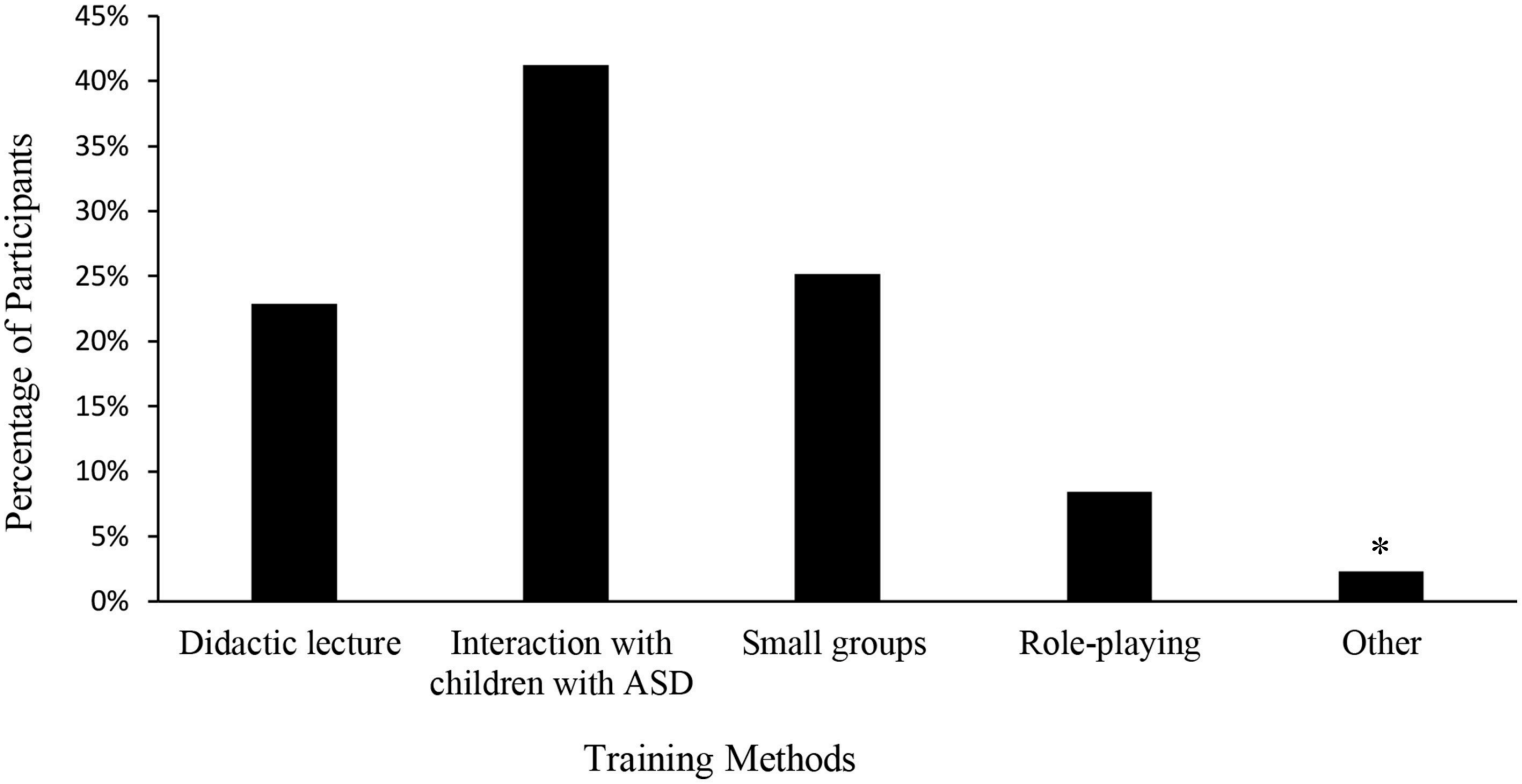
Perceived Best Training Method of Learning by Medical Students and Trainees. ^*^Other: working with parents of children with ASD, learning from other subspecialties such as occupational therapists, and special courses in medical school.

91.4% of medical students and pediatric trainees reported that they did not received enough didactic or clinical training regarding treatment of a child with ASD when they present with an acute illness, and 92.6% felt that more training on this topic was warranted. When queried regarding ideal training methods, most selected interaction with children with ASD (162, 41.2%), followed by small group sessions (99, 25.2%), didactic lecture (90, 22.9%), role-playing (33, 8.4%), and other methods (9, 2.3%) (Figure 4). Other methods included working with parents of children with ASD, learning from other subspecialties such as occupational therapists, and special courses in medical school designed around this topic. Lastly, a higher number of female participants believed that they needed more training on children with ASD (96.3 vs. 87.0%, *p* = 0.0235).

## Discussion

With the increasing prevalence of ASD (11), it is critical that healthcare providers have a solid foundation on issues unique to these patients, particularly when they present with an acute medical illness. In this single center study, assessing the perceived general knowledge of ASD in medical students and pediatric trainees, we found that in general, all learners reported a lack of knowledge, had a perceived discomfort in managing medically ill children with ASD, coupled with a desire for more training in this space.

Overall, the perceived general knowledge of ASD was low in both medical students and pediatric trainee (Table 2). However, the pediatric trainees reported more knowledge than the medical students. More training, education, and exposure on the topic and patients most likely accounted for the increase in knowledge seen in the pediatric trainees. Without emphasizing this common disease state in the curriculum, we risk misunderstanding of presentations and failures of communication on the wards that may be detrimental to the care process of this increasingly recognized condition.

Children with ASD are partial to routine and order. Disruption of a daily routine can cause a child to become distressed, making it more difficult to communicate with him or her (12). Both medical students and pediatric trainees rated the importance of understanding the baseline behavior of a child with ASD high, with a higher rating given by the pediatric trainees. Similarly, both groups rated the importance of daily routine for ASD children as high. It is imperative that clinicians understand the baseline behavior of their patients, as well as having an appreciation of the importance in routine disruption (13). This is especially important in an acute care setting such as the ER where patients may be interacting for the first time with specific providers. Frontline physicians need to make the assessment for baseline behaviors (often can be elicited via directed questions during history taking) a necessary step in their management of the acutely ill child.

Communication can often become a barrier in providing care to children with ASD. In a recent study, Solomon et al reported that clinicians often rarely spoke to their pediatric patients with ASD during primary or acute care visits (14). In another study, Nicolaidis et al. found that adults with ASD often reported lower satisfaction rates with the quality of communication with their healthcare provider, when compared to neuro-typical adults without ASD (15). Understanding non-verbal communication, and effective use of this communication method is important as it may potentially facilitate better overall care for patients with ASD (16–18). Examples of non-verbal communication includes use of drawings, exaggerated hand gestures, use of full body movements and appropriate props (16, 17). In our surveyed population, more than 50% of medical students and pediatric trainees elected using visual aids as the preferred method of communication with a child with ASD. However, approximately a quarter of the surveyed participants selected verbal communication as the preferred method, which may potentially pose a challenge since verbal communication is often not an effective communication tool for patients with ASD (Figure 1). Taken together, our findings suggest that additional training in optimal methods of communication is needed among the surveyed trainees. This training will be a critical piece of the skill set necessary for successfully managing acute illness in children with ASD.

Sensory dysregulation is an important core feature of children with ASD (19–21). However, in our surveyed population, although the pediatric trainees reported a higher understanding of sensory regulation compared to medical students, the mean rating was overall low at 5 (out of 10, 10 being the highest rating score; Figure 2). Compared to pediatric trainees, medical students rated the frequency of sensory dysregulation in children with ASD to be lower (Figure 3). This finding highlights the need for increased awareness of the implications of children with ASD having sensory challenges, particularly when they present with an acute illness (22, 23). In a pilot study of a community zoo, implementation of a sensory training program resulted in more visitations to the zoo by children with ASD and a better overall experience for the child, families, and staff members (24). Staff training included ways to identify a guest with sensory sensitivity, methods of communication and engagement, as well as techniques to handle situations when a child was in sensory overload. It may be posited that a training program with a focus on sensory processing challenges may be beneficial for medical providers. Such a program will increase the understanding of sensory sensitivities that are often heightened during acute illness and will provide communication techniques, as well as potentially allowing for tangible modification of the environment (for instance provision of sensory tools such as noise canceling headphones). The goal is ultimately to mitigate these sensory barriers that may hinder appropriate and timely diagnosis or management, as well as improve patient satisfaction and service.

Clinicians in general report an increased feeling of discomfort when having encounters or managing patients with ASD (24–29). In our study, more than 90% of medical students and pediatric trainees reported insufficient didactic and clinical training for managing a child with ASD who presents with an acute illness. Similarly, more than 90% reported the need for increased training in this arena. Interestingly, there was no difference across training levels in the reported need for more training, with pediatric trainees reporting the need just as frequently as first year medical students. Across all training levels, interaction with children with ASD was the most frequently selected option for best method of teaching, followed by small group learning (Figure 4). Others have shown that facilitated direct encounters with children with ASD could potentially increase the comfort level of trainees for future encounters with ill children with ASD who present in a hospital setting (30, 31). Creating a pediatric training curriculum centered around patient interactions and small group learning could provide the knowledge necessary in a format preferred by students, to properly treat children with ASD when they present with acute illnesses (32, 33).

In this single center study, we identified several limitations. First, the sample size is small as the research was conducted in one academic medical institution and the partnering children's hospital. A survey bias could have occurred due to convenience sampling, and the overall low response rate from certain groups, therefore affecting the potential generalizability of the results. However, these initial data are important as they will inform the design of a large prospective study involving not just physicians but other providers such as nurses, that will provide more granularity to the deficits identified and how widespread they are. The participants were also not evenly distributed among each level of training. In our analysis, medical students were grouped and compared with pediatric trainees. Because most of the medical students (33.5%) were from the first year, it did naturally allow for comparison between the lowest level trainee (first year medical student), and the highest-level trainees (pediatric fellows). Lastly, the questions asked on this survey were not exhaustive to the information that could have been collected. More areas of limited understanding on ASD may exist but were not covered in this study.

## Conclusion

The findings of this study suggest that medical students and pediatric trainees across all levels perceived a lack of knowledge regarding ASD. Medical students and pediatric trainees also reported increased discomfort and need for increased education and training in relation to the treatment of children with ASD who present with an acute illness. Taken together, this study highlights current gaps and underscores the importance of ensuring adequate knowledge and training for medical professionals particularly as it relates to improving our ability to care for medically ill patients with ASD.

## Ethics Statement

This study was performed with the University of Alabama at Birmingham institutional review board (IRB) approval (Protocol E160414004) and carried out with voluntary and electronic informed consent from all study participants. Survey respondent anonymity was ensured by not collecting any personally identifiable information. All data were stored in a password-protected server.

## Author Contributions

KA created the survey, collected and analyzed the data, and contributed toward the writing of the manuscript. IA, JW, and MK analyzed and interpreted survey results and contributed toward the writing of the manuscript. All authors approved the final manuscript.

### Conflict of Interest Statement

The authors declare that the research was conducted in the absence of any commercial or financial relationships that could be construed as a potential conflict of interest.
