# Supplementary material for: Contemporary Trainee Knowledge of Autism: How Prepared Are Our Future Providers?
Source: Front Pediatr. 2019 Apr 26;7:165. doi: 10.3389/fped.2019.00165 (PMC6498887; doi:10.3389/fped.2019.00165)
Supplement: Supplementary file 1 [file Data_Sheet_1.docx]

Appendix:

**Survey Questionnaire**

1. Please describe your highest educational level.

a. MS1

b. MS2

c. MS3

d. MS4

e. Pediatric Intern

f. Pediatric resident

g. Pediatric fellow

2. Please describe your age.

a. 21-30

b. 31-40

c. 41-50

d. 50+

3. Please describe ethnicity.

a. Caucasian

b. Africa-American

c. Asian

d. Hispanic

e. Other:

4. Please describe your gender.

5. How would you describe your general knowledge of children with Autism Spectrum Disorder (ASD)?

a. Very informed

b. Somewhat informed

c. Limited information

d. No information

6. How would you describe your interaction with children with ASD?

a. Daily interaction

b. Weekly interaction

c. Monthly interaction

d. Limited interaction

e. No interaction

7. If you have interactions with children with ASD on a monthly basis or more, please describe below the environment in which this occurs. Check all that apply.

a. Not applicable

b. Medical

c. Familial

d. Work environment other than in a medical setting

e. Other:

8. If you have interactions with children with ASD on a monthly basis or more, please describe your method of communication. Check all that apply.

a. Not applicable

b. Speaking

c. Visual aids

d. Hand motions

e. Eye contact

f. Written word

g. Other:

9. How do you believe children with ASD communicate the best with people around them? Check all that apply.

a. Speaking

b. Visual aids

c. Hand motions

d. Eye contact

e. Written word

f. Other:

10. What do you think is the best method to communicate with children with ASD?

a. Speaking

b. Visual aids

c. Hand motions

d. Eye contact

e. Written word

f. Other:

11. How important do you think it is to understand a child with ASD’s daily behavior? Please rate on a scale of 1-10, with 1 being the least important and 10 being the most important.

12. How important do you think it is to understand a child with ASD’s daily routine? Please rate on a scale of 1-10, with 1 being the least important and 10 being the most important.

13. Have you ever provided care for a child with ASD who presented with an acute illness?

a. Yes

b. No

14. If you answered yes to question 13, where was the encounter? If you answered no, please select not applicable.

a. Clinic

b. Emergency Room

c. In Patient Hospital

d. Not applicable

15. How comfortable do you feel providing care for a child with ASD who presents with an acute illness? Please rate on a scale of 1-10, with 1 being the least comfortable and 10 being the most comfortable.

16. How do you believe children with ASD communicate the best with people around them when they present with an acute illness? Check all that apply.

a. Speaking

b. Visual aids

c. Hand motions

d. Eye contact

e. Written word

f. Other:

17. What do you think is the best method to communicate with children with ASD who present with an acute illness?

a. Speaking

b. Visual aids

c. Hand motions

d. Eye contact

e. Written word

f. Other:

18. How important do you think it is to ask the family about the child’s routine when the child presents with an acute illness? Please rate on a scale of 1-10, with 1 being the least important and 10 being the most important.

19. What do you think is the frequency of a child with ASD experiencing sensory dysregulation? Please rate on a scale of 1-10, with 1 being not often and 10 being very often.

20. What do you think is your understanding of sensory issues in children with ASD? Please rate on a scale of 1-10, with 1 being very limited and 10 being very well understood.

21. Do you think you receive enough didactic or clinical training regarding treatment of a child with ASD who presents with an acute illness?

a. Yes

b. No

22. Do you think you need more training on this topic?

a. Yes

b. No

23. What do you think is the best way to teach/train on this topic? Check all that apply.

a. Didactic lecture

b. Interaction with children with ASD

c. Small groups

d. Role-playing

e. Other:
